# Attractor dynamics in local neuronal networks

**DOI:** 10.1186/1471-2202-14-S1-P386

**Published:** 2013-07-08

**Authors:** Rosa Comas, André Longtin, Jean-Philippe Thivierge

**Affiliations:** 1 School of Psychology, University of Ottawa, Ottawa, Ontario K1N 6N5, Canada; 2 Department of Physics, University of Ottawa, Ottawa, Ontario K1N 6N5, Canada

## 

A hallmark feature of cortical networks is the presence of synaptic motifs, defined as ensembles of neurons whose synaptic pattern follows a particular configuration [1]. Simulated networks of neurons whose excitatory synapses follow a three-node "relay" motif (Figure 1A)-the most frequent motif in primate visual cortex- exhibit synchronization with zero time lag [2], a form of activity reported in a spectrum of experiments [3]. Here, using simulations of leaky integrate-and-fire networks (LIF) as well as mean-field stability analyses, we show that this relay motif promotes the emergence of a limit cycle whose period is determined by intrinsic properties of the model (Figure 1B). While cortical recordings show evidence of limit-cycle oscillations [4], this behavior is typically transient in non-pathological states. The question thus arises, of how to generate transient yet precise synchronization under different forms of motif connectivity. To address this question, we introduce a mechanism of *selective gain inhibition *by which cortical circuits may disengage from a strict limit cycle behavior. This mechanism works by tuning the gain inhibition [5] of a selective population of neurons in the model. In a first series of simulations, we show that applying selective gain inhibition to one population of a network (Figure 1A, shown in black) disengages the network from a limit cycle behaviour (Figure 1C). Next, we examine the effect of selective gain inhibition on a network's response to an incoming stimulus and show that transient synchronization arises in response to a time-delimited input current (Figure 1D). Selective gain inhibition enables stimulus-induced synchronization under strong stimulation and suppresses zero-lag synchrony under weak stimulation (Figure 1E). Transient synchronization would not be possible without selective gain inhibition, given that a network configured with a "relay" motif follows a limit cycle attractor (Figure 1B). We conclude that a "relay' motif of connectivity imposes strict constraints on the types of dynamics produced by a network under both spontaneous and evoked states. Going further, results of simulations suggest that a mechanism of selective gain inhibition breaks the rigid constraints imposed by synaptic connectivity, providing flexible and transient responses to incoming stimuli.

**Figure 1:**
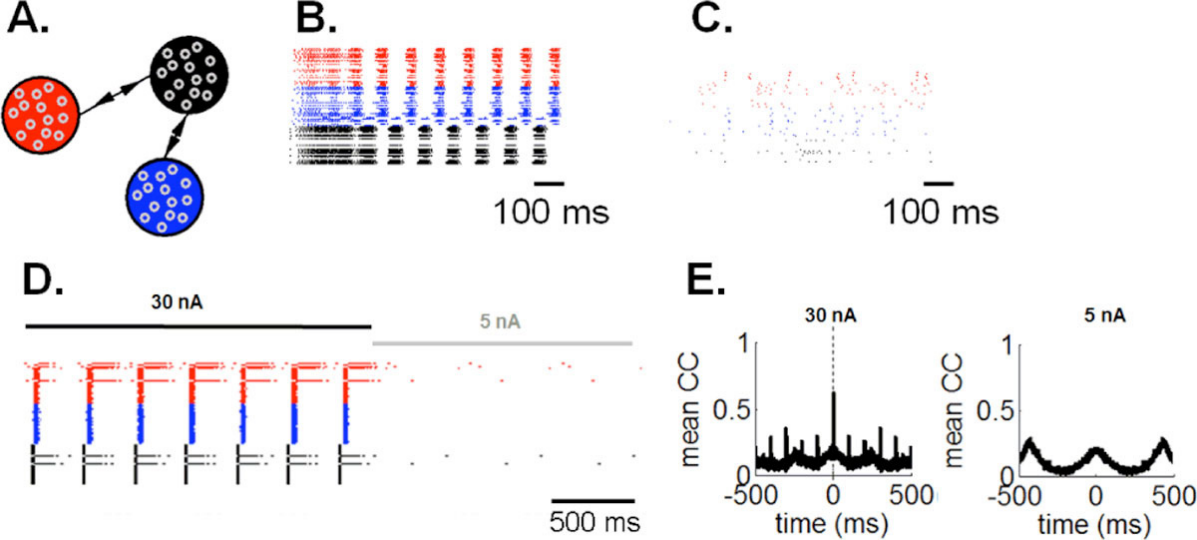
**Transient synchronization in LIF networks**. **A**. Three groups of neurons with arrows showing the presence of between-group synapses (1,000 neurons/group; gain inhibition set to 1.5 nS). **B**. Spike raster of spontaneous activity for network in A. **C**. Raster with gain inhibition of neuron group in black set to 2.25 nS). **D**. Same as C, with all excitatory neurons injected with a constant current. **E**. Cross-correlations averaged over all neurons, with constant current (each simulation lasting 10 sec).

## References

[B1] Song S Sjostrom PJ Reigl M Nelson S Chklovskii DB Highly nonrandom features of synaptic connectivity in local cortical circuits PLoS Biol 2005 3 3 e68 10.1371/journal.pbio.0030068 15737062 PMC1054880

[B2] Vicente R Gollo LL Mirasso CR Fischer I Pipa G Dynamical relaying can yield zero time lag neuronal synchrony despite long conduction delays Proc Natl Acad Sci USA 2008 105 44 17157 17162 10.1073/pnas.0809353105 18957544 PMC2575223

[B3] Benda J Longtin A Maler L A synchronization-desynchronization code for natural communication signals Neuron 2006 52 2 347 358 10.1016/j.neuron.2006.08.008 17046696

[B4] Rodriguez E George N Lachaux JP Martinerie J Renault B Varela FJ Perception's shadow: long-distance synchronization of human brain activity Nature 1999 397 6718 430 433 10.1038/17120 9989408

[B5] Vogels TP Abbott LF Gating multiple signals through detailed balance of excitation and inhibition in spiking networks Nat Neurosci 2009 12 4 483 491 10.1038/nn.2276 19305402 PMC2693069

